# Supplementary material for: A pathogen effector HaRxL10 hijacks the circadian clock component CHE to perturb both plant development and immunity
Source: Nat Commun. 2025 Feb 11;16:1538. doi: 10.1038/s41467-025-56787-w (PMC11814294; doi:10.1038/s41467-025-56787-w)
Supplement: Supplementary file 1 — Supplementary Information [file 41467_2025_56787_MOESM1_ESM.pdf]

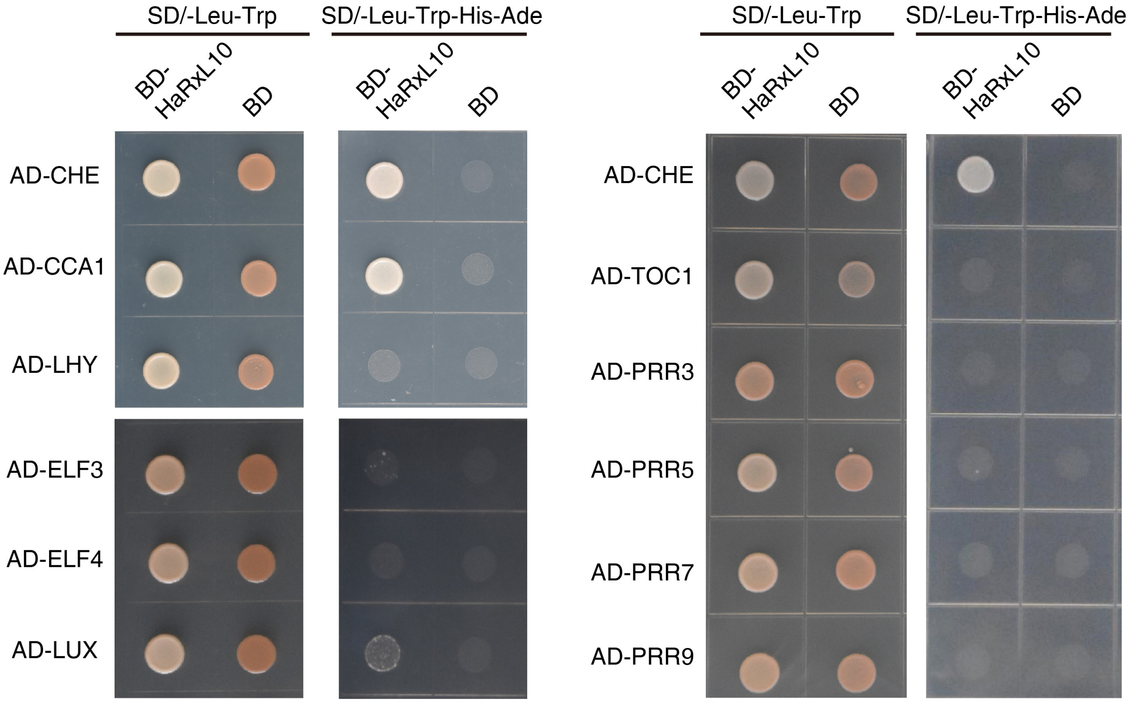

Supplementary Fig. 1 | Interaction between HaRxL10 and central clock components in yeast. Synthetic dropout medium without leucine and tryptophan (SD/-Leu-Trp) was used for positive yeast transformant selection. Synthetic dropout medium without leucine, tryptophan, histidine and adenine (SD/-Leu-Trp-His-Ade) was used for the selection of protein interaction by the reporter gene *HIS3*. Photographs were taken 2 days after plating of yeast cells with $OD_{600\ nm}$ = 1. This experiment was repeated three times with similar results.

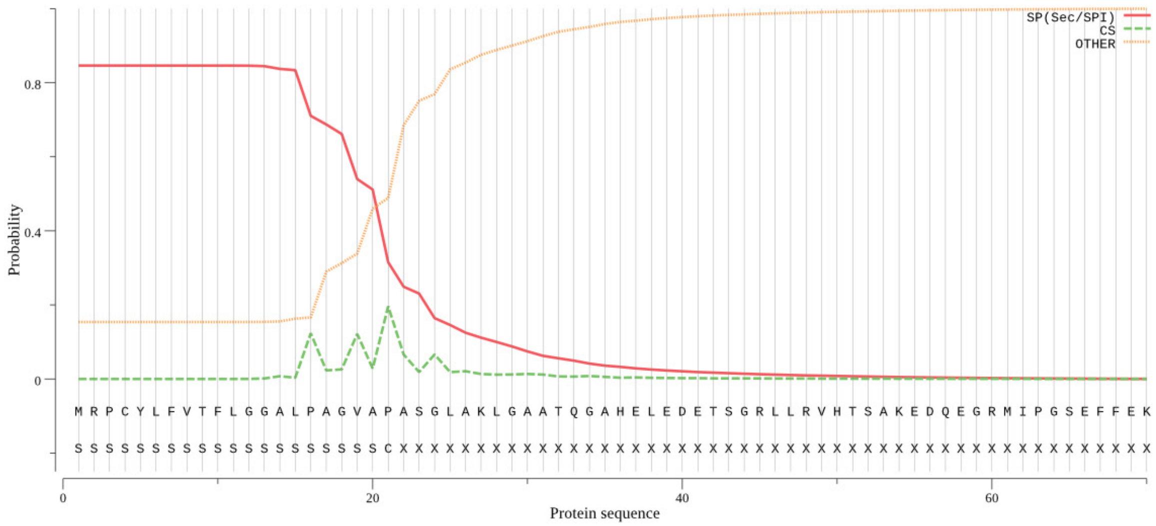

Supplementary Fig. 2 | Prediction of the signal peptide of HaRxL10. Prediction of the signal peptide sequence of HaRxL10 using the SignalP-5.0 website (https://services.healthtech.dtu.dk/services/SignalP-5.0/). The predicted cleavage site is between position 21 and 22 amino acids.

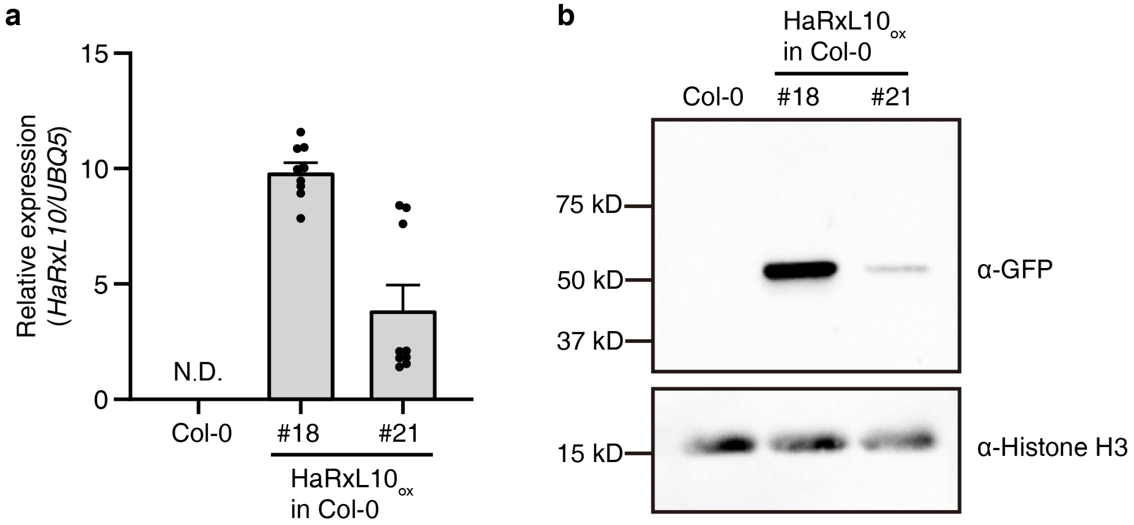

Supplementary Fig. 3 | Validation of HaRxL10 overexpression transgenic lines. a Relative gene expression of *HaRxL10* in the wild-type (Col-0) and two independent HaRxL10 overexpression (*35S:YFP-HaRxL10*, HaRxL10$_{ox}$) lines analysed by RT-qPCR with *UBQ5* as an internal control. The data are shown as mean ± SEM (n = 9, 3 independent experiments with 3 technical replicates). N.D., not detected. **b** Representative Western blot images showing the protein levels of YFP-HaRxL10 in the wild-type (Col-0) and two independent HaRxL10 overexpression (*35S:YFP-HaRxL10*, HaRxL10$_{ox}$) lines. Histone H3 was used as an internal control. The molecular weight of YFP-HaRxL10 is 52.3 kD. The molecular weight of Histone H3 is 15 kD. This experiment was repeated three times (**b**) with similar results.

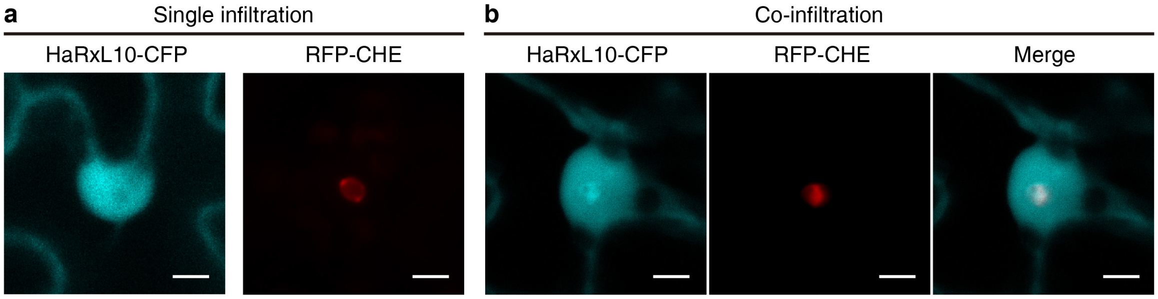

Supplementary Fig. 4 | Subcellular localisation of HaRxL10 and CHE. Representative images of subcellular localisation of HaRxL10-CFP and RFP-CHE proteins when individually expressed (**a**) or co-expressed (**b**) in tobacco leaves. Scale bars, 5 μm. This experiment was repeated three times with similar results.

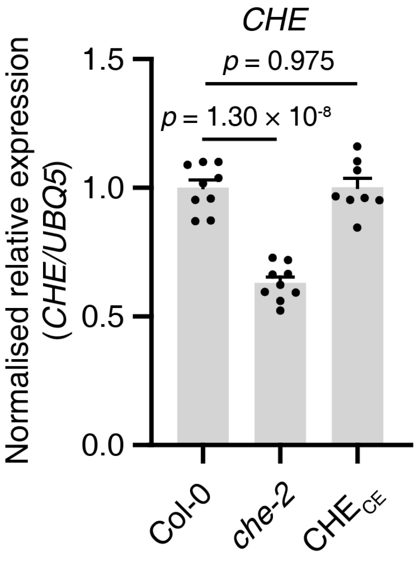

Supplementary Fig. 5 | Validation of CHE complementation line. The transcript levels of *CHE* in the wild-type (Col-0), *che-2*, and CHE complementation (CHE_{CE}, *CHEp:CHE-4×Myc* in *che*-2) were analysed by RT-qPCR with *UBQ5* as an internal control and normalised by the expression in Col-0. Samples were collected at ZT8, which is the peak expression time of *CHE*. The data are shown as mean ± SEM (n = 9, 3 independent experiments with 3 technical replicates). The *p* values were calculated by one-way ANOVA followed by Holm-Šídák's multiple comparisons test.

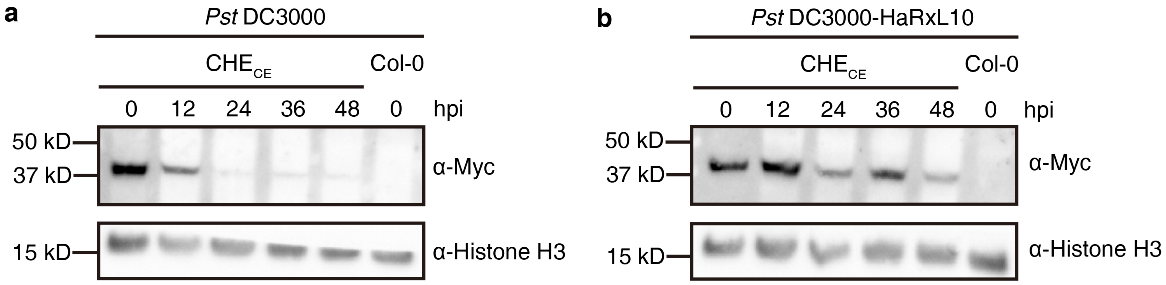

Supplementary Fig. 6 | HaRxL10 inhibits *Pst* DC3000-induced decrease in the protein abundance of CHE. Representative Western blot images showing CHE-Myc protein abundance. Three-week-old *Arabidopsis* leaves were inoculated with *Pst* DC3000 (**a**) or *Pst* DC3000-HaRxL10 (**b**) ($OD_{600\ nm}$ = 0.002) and samples were collected at 0, 12, 24, 36, and 48 hours post-infiltration (hpi), respectively. CHE_{CE}, *CHEp:CHE-4×Myc* in *che-2*. Col-0 was used as a negative control. The molecular weight of CHE-4×Myc is 32.4 kD. The molecular weight of Histone H3 is 15 kD. These experiments were repeated two times with similar results.

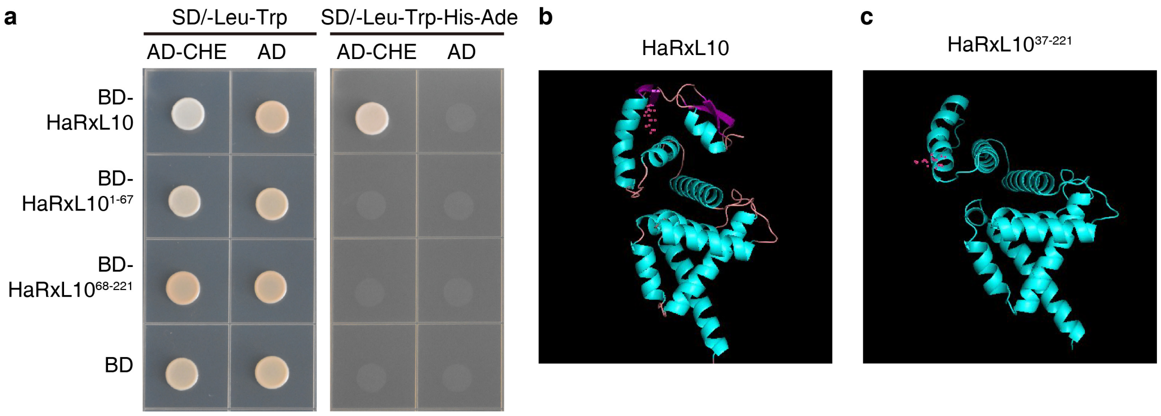

Supplementary Fig. 7 | Characteristics of truncated HaRxL10 proteins. a Y2H assays showing that the truncated HaRxL10 proteins, $HaRxL10_{1\text{-}67}$ and $HaRxL10^{68\text{-}221}$ could not interact with CHE in yeast. This experiment was repeated three times with similar results. **b, c** The 3D structures of full-length HaRxL10 (**b**) and $HaRxL10^{37\text{-}221}$ (**c**) predicted by AlphaFold2 and illustrated by PyMOL.

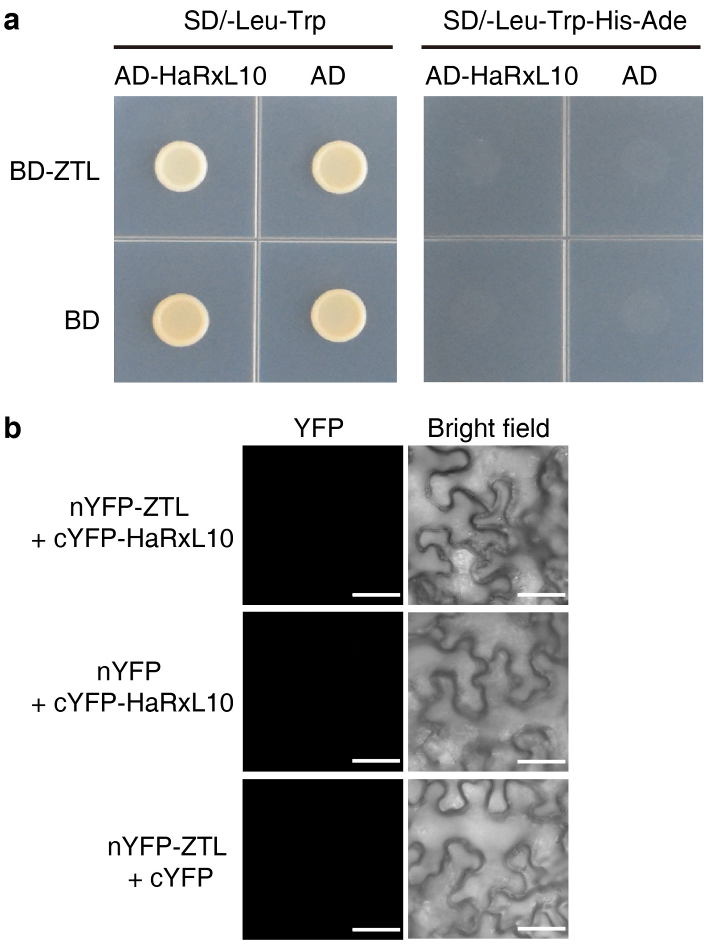

Supplementary Fig. 8 | HaRxL10 does not interact with ZTL in either yeast or plant cells. a The Y2H experiments demonstrating that HaRxL10 does not interact with ZTL in the yeast system. Photographs were taken 2 days after plating of yeast cells on selection plates with $OD_{600\ nm}$ = 1. **b** BiFC experiments illustrating that HaRxL10 does not interact with ZTL in *N. benthamiana*. Proteins were expressed in tobacco for 2 days and then observed under a confocal microscope. Scale bars, 10 μm. These experiments were repeated three times (**a and b**) with similar results.

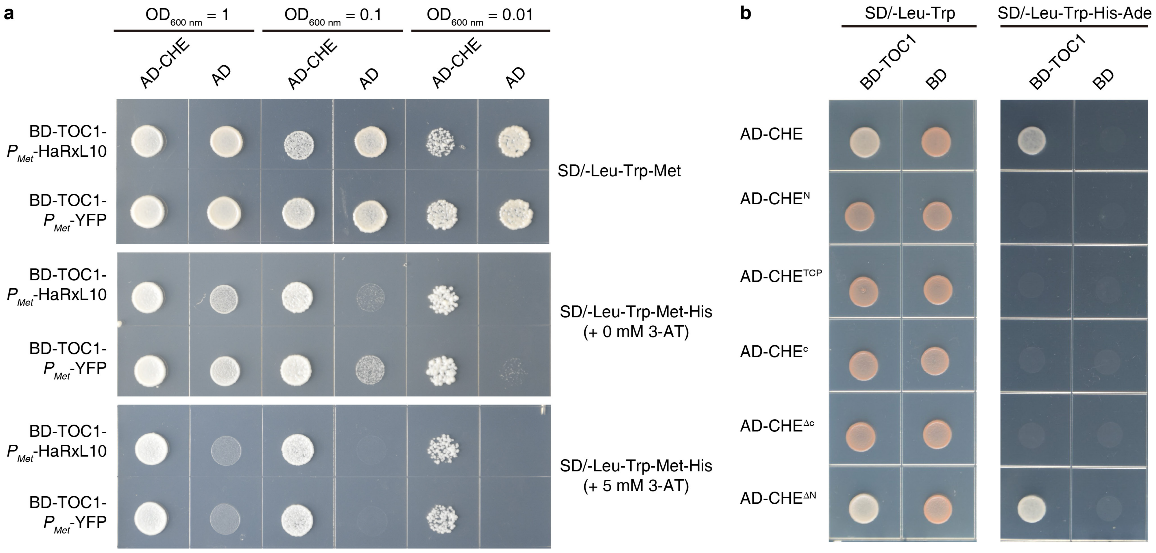

Supplementary Fig. 9 | HaRxL10 does not interfere with the interaction between CHE and TOC1 in yeast. a Y3H experiments illustrating that HaRxL10 does not interfere with the interaction between CHE and TOC1 in yeast. 3-AT was used to inhibit the self-activation in yeast. Photographs were taken 3 days after plating yeast cells on selection plates with different concentrations of yeast cells and 3-AT. **b** Y2H experiments demonstrating that TOC1 interacts with full-length CHE and N-terminal truncated CHE ($CHE^{\Delta N}$) in yeast. Photographs were taken 2 days after plating yeast cells on selection plates with $OD_{600\ nm}$ = 1. These experiments were repeated three times (**a and b**) with similar results.

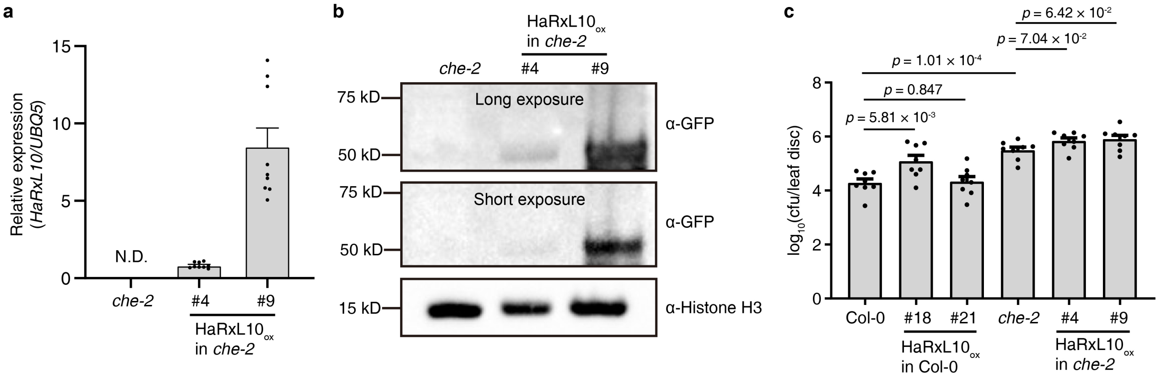

Supplementary Fig. 10 | The virulence of HaRxL10 is dependent on CHE. a Relative gene expression of *HaRxL10* in the *che-2* and two independent HaRxL10 overexpression (*35S:YFP-HaRxL10* in *che-2*) lines analysed by RT-qPCR with *UBQ5* as an internal control. The data are shown as mean ± SEM (n = 9, 3 independent experiments with 3 technical replicates). N.D., not available. **b** Representative Western blot images showing the protein levels of YFP-HaRxL10 in *che-2* and two independent HaRxL10 overexpression (*35S:YFP-HaRxL10* in *che-2*) lines. Histone H3 was used as an internal control. The molecular weight of YFP-HaRxL10 is 52.3 kD. The molecular weight of Histone H3 is 15 kD. **c** Bacterial growth in 3-week-old *Arabidopsis* leaves infiltrated with *Pst* DC3000 ($OD_{600\ nm}$ = 0.00005) at 3 days post-infiltration. Data represent the mean ± SEM (n = 8 plants). The *p* values were calculated by one-way ANOVA followed by Holm-Šídák's multiple comparisons test. cfu, colony forming unit. These experiments were repeated three times (**b and c**) with similar results.

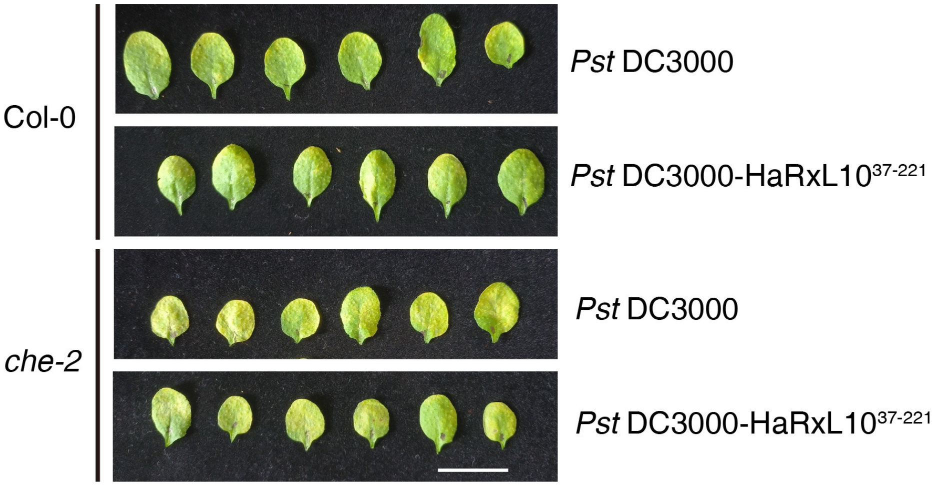

Supplementary Fig. 11 | HaRxL10$^{37\text{-}221}$ enhances plants disease susceptibility to *Pst* DC3000. Disease symptoms in wild-type (Col-0) and *che-2* *Arabidopsis* leaves infiltrated with *Pst* DC3000 or *Pst* DC3000-HaRxL10$^{37\text{-}221}$ ($OD_{600\ nm}$ = 0.002) at 3 days post-infiltration. Scale bar, 2 cm.

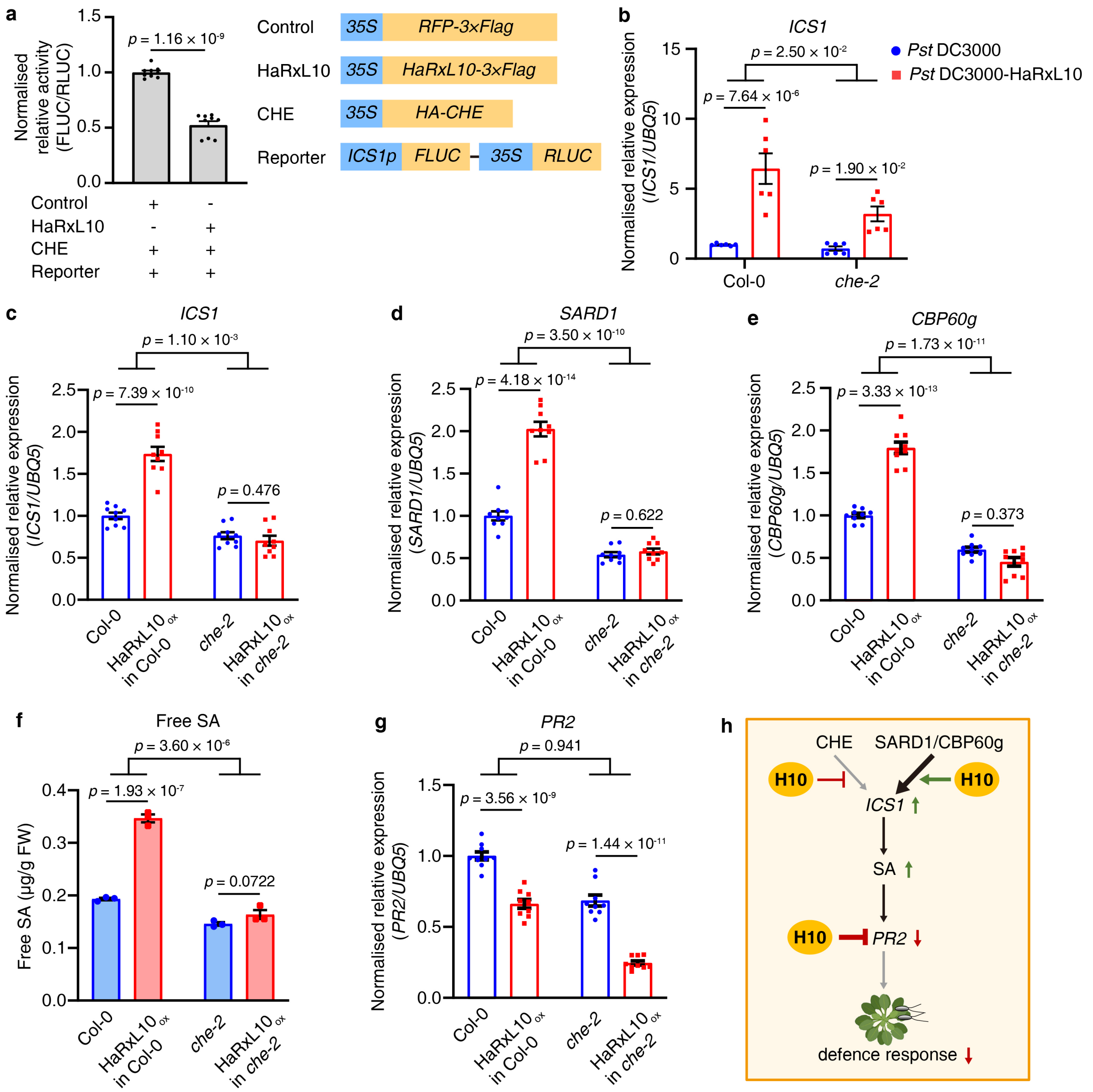

Supplementary Fig. 12 | Effects of HaRxL10 on SA-related gene expression and SA levels. a Dual-luciferase assay performed using *N. benthamiana* leaves transiently co-expressing different protein combinations and the reporter driven by *ICS1* promoter. The ratio of firefly luciferase (FLUC) and *Renilla* luciferase (RLUC) activities was calculated and normalised to the control. The data are shown as mean ± SEM (n = 9, 3 independent experiments with 3 technical replicates). The *p* value was calculated by two-sided unpaired Student's *t*-test. **b** Relative gene expression levels of *ICS1* in the wild-type (Col-0) and *che-2 Arabidopsis* plants at 24 hours after *Pst* DC3000 or *Pst* DC3000-HaRxL10 ($OD_{600\ nm}$ = 0.002) infection analysed by RT-qPCR with *UBQ5* as an internal control. The data are shown as mean ± SEM (n = 6, 2 independent experiments with 3 technical replicates). The *p* values were calculated by two-way ANOVA followed by Šídák's multiple comparisons test. **c-g** SA-related gene expression and endogenous SA levels were analysed in 3-week-old *Arabidopsis* leaves infiltrated with *Pst* DC3000 (OD_{600}

$_{nm}$ = 0.00005) at 1 day post-infiltration. **c, d, e, g** Relative expression of *ICS1* (**c**), *SARD1* (**d**), *CBP60g* (**e**) and *PR2* (**g**) analysed by RT-qPCR with *UBQ5* as an internal control. The expression levels were normalised to the relative expression level in the wild-type plants. Data represent the mean ± SEM (n = 9, 3 independent experiments with 3 technical replicates). The *p* values were calculated by two-way ANOVA followed by Šídák's multiple comparisons test. **f** Endogenous free SA levels were measured. Data represent the mean ± SEM (n = 3 independent experiments). The *p* values were calculated by two-way ANOVA followed by Šídák's multiple comparisons test. **h** Illustration of effects of HaRxL10 (H10) on SA-related gene expression and SA levels. HaRxL10 has multiple targets and exerts different effects on different targets. HaRxL10 represses CHE-mediated *ICS1* induction but promotes SARD1/CBP60g-mediated *ICS1* induction, resulting in higher *ICS1* expression and SA levels. HaRxL10 also represses *PR2* expression to dampen defence response and cause disease susceptibility.

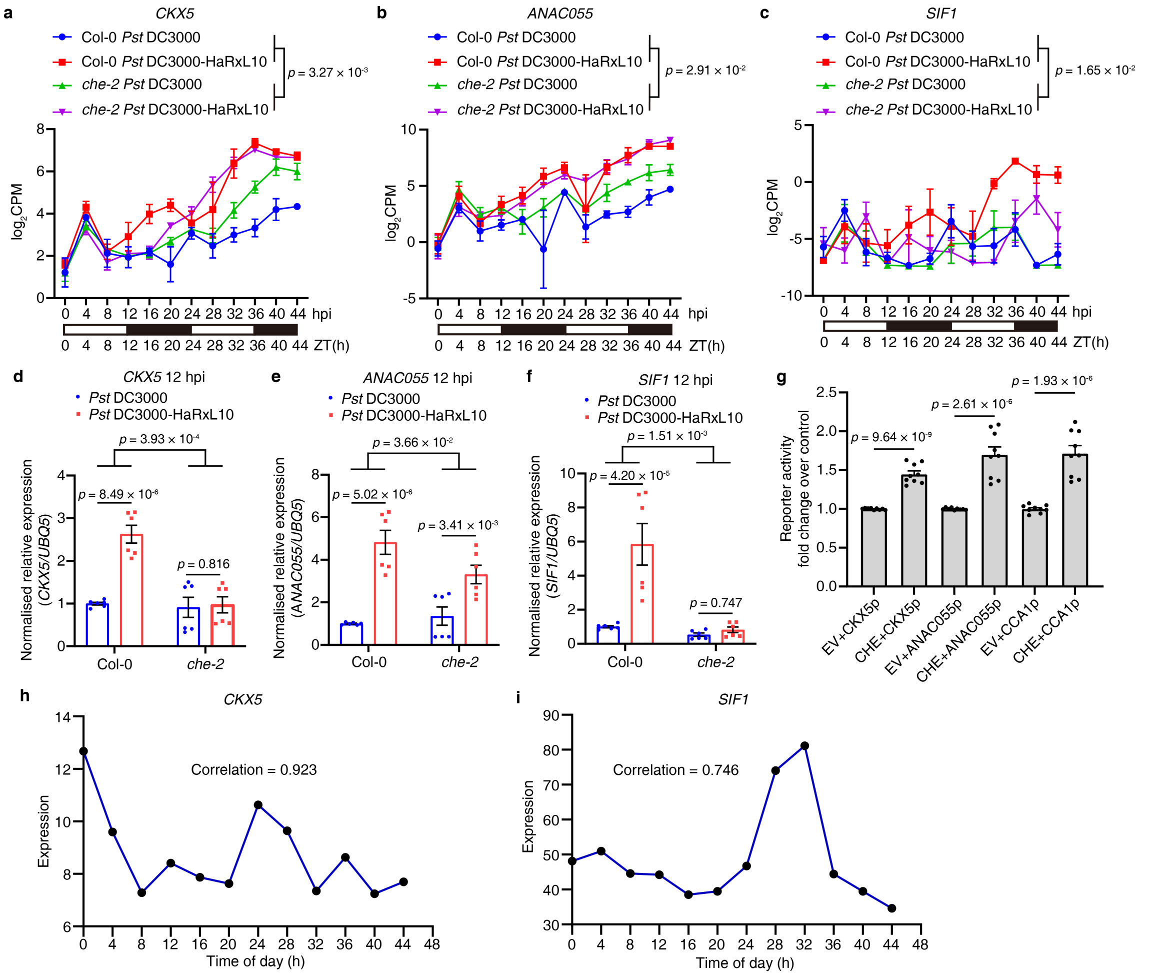

Supplementary Fig. 13 | CHE regulates HaRxL10-mediated induction of *CKX5*, *ANAC055*, and *SIF1*. a-c Transcript abundance of *CKX5* (**a**), *ANAC055* (**b**), and *SIF1* (**c**) in the wild-type (Col-0) *Arabidopsis* leaves after infiltrated with *Pst* DC3000 or *Pst* DC3000-HaRxL10 represented by $\log_2$CPM (count-per-million) from time-course RNA-seq dataset. The data are shown as mean ± SEM (n = 3 biological replicates). The *p* values were calculated by linear models followed by empirical Bayesian analysis on the two-way interaction between the presence of CHE and HaRxL10. hpi, hour post-infiltration. White bar, light. Black bar, dark. ZT, Zeitgeber time. **d-f** Relative gene expression of *CKX5* (**d**), *ANAC055* (**e**), and *SIF1* (**f**) in the wild-type (Col-0) and *che-2 Arabidopsis* plants at 12 hours after *Pst* DC3000 or *Pst* DC3000-HaRxL10 infection analysed by RT-qPCR with *UBQ5* as an internal control. The data are shown as mean ± SEM (n = 6, 2 independent experiments with 3 technical replicates). The *p* values were calculated by two-way ANOVA followed by Holm-Šídák's multiple comparisons test. **g** Y1H assays demonstrated that CHE could bind to the promoters of *CKX5* and *ANAC055*. $OD_{420\ nm}$ was measured and β-galactosidase reporter activities are shown as fold change of CHE over AD empty vector (EV). The data are shown as mean ± SEM (n = 9, 3 independent experiments with 3 technical replicates). The *p* values were calculated using two-sided unpaired Student's *t*-test. The promoter of *CCA1* was

used as a positive control. **h, i** Expression profiles of *CKX5* (**h**) and *SIF1* (**i**) under the circadian condition retrieved from the Diurnal database (http://diurnal.mocklerlab.org/diurnal_data_finders/new). Plants were entrained by light/dark cycles and gene expression was analysed under the constant light condition.

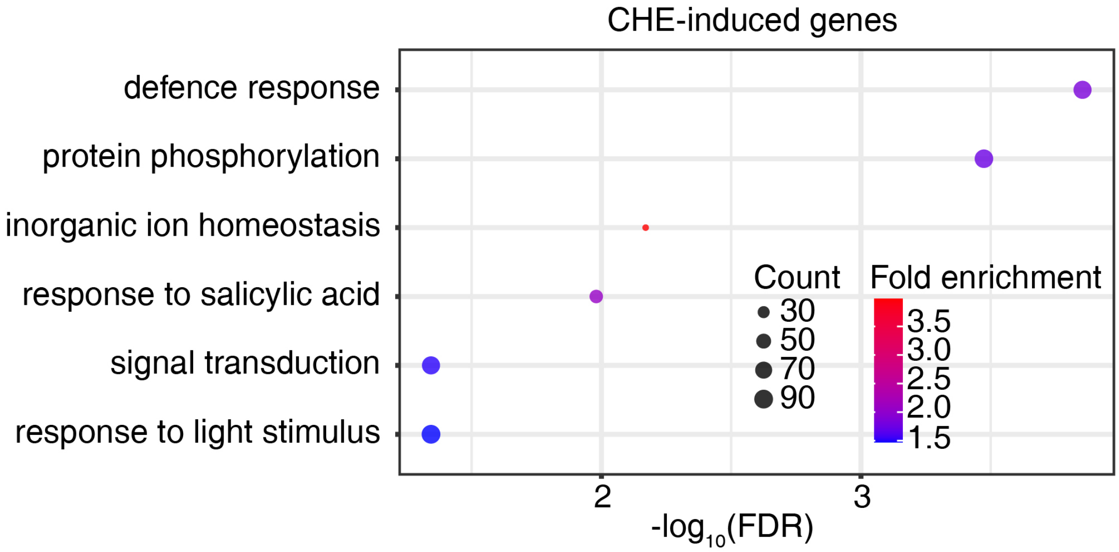

Supplementary Fig. 14 | Enriched GO terms of biological processes among CHE-induced genes. Enriched GO terms of biological processes among CHE-induced genes identified through RNA-seq analysis of the wild-type (Col-0) and *che-2* upon *Pst* DC3000 infiltration. FDR, false discovery rate.

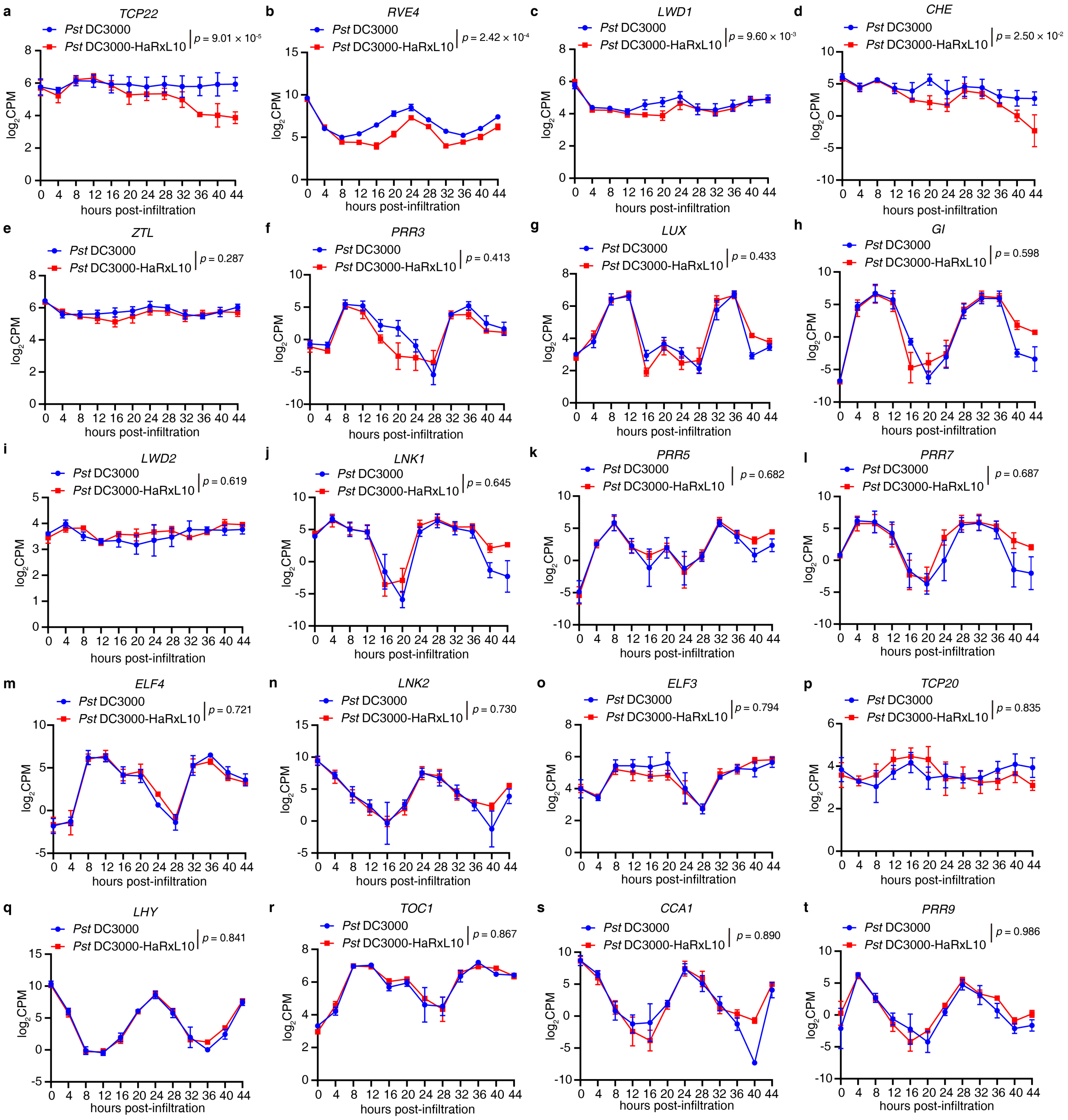

Supplementary Fig. 15 | Effects of HaRxL10 on transcription levels of *Arabidopsis* central clock genes. Transcript abundance of 20 central circadian clock genes in the wild-type (Col-0) *Arabidopsis* leaves after infiltrated with *Pst* DC3000 or *Pst* DC3000-HaRxL10 represented by log_2CPM (count-per-million) from time-course RNA-seq dataset. The data are shown as mean ± SEM (n = 3 biological replicates). The *p* values were calculated by linear models followed by empirical Bayesian analysis on the contrast between *Pst* DC3000 and *Pst* DC3000-HaRxL10.

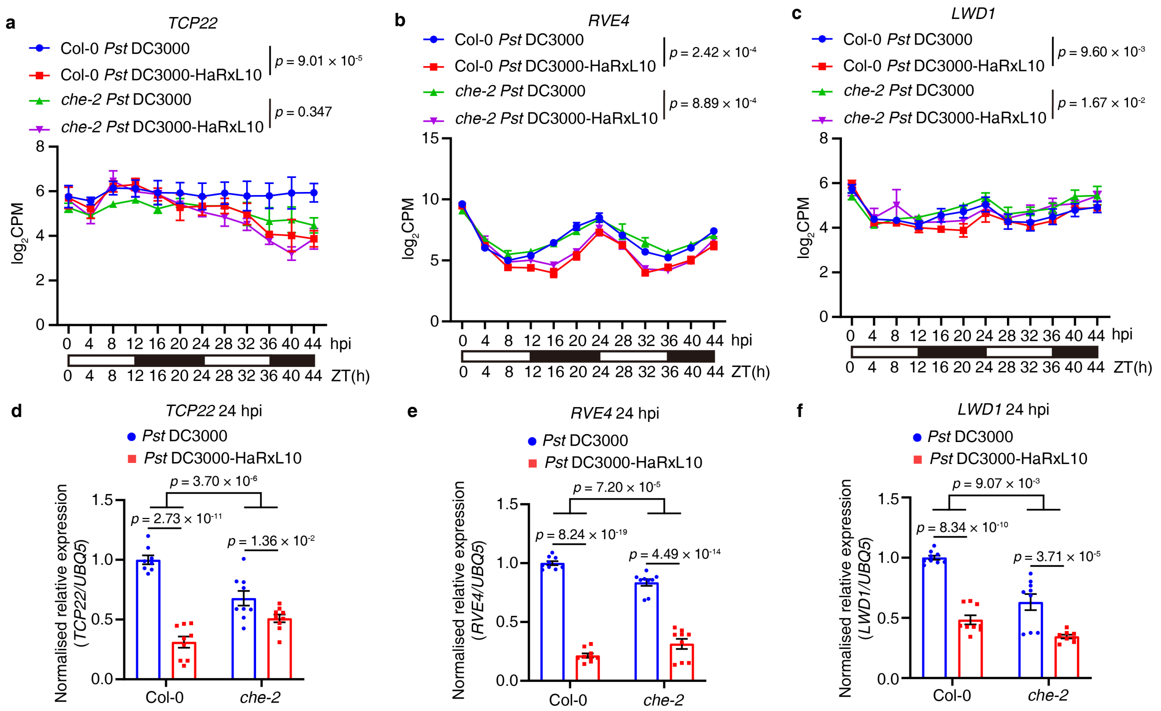

Supplementary Fig. 16 | HaRxL10 affects the expression of several clock genes. a-c Transcript abundance of *TCP22* (**a**), *RVE4* (**b**), and *LWD1* (**c**) in the wild-type (Col-0) and *che-2 Arabidopsis* leaves after infiltrated with *Pst* DC3000 or *Pst* DC3000-HaRxL10 represented by log_2CPM (count-per-million) from time-course RNA-seq dataset. The data are shown as mean ± SEM (n = 3 biological replicates). The *p* values were calculated by linear models followed by empirical Bayesian analysis on the indicated contrasts. hpi, hour post-infiltration. White bar, light. black bar, dark. ZT, Zeitgeber time. **d-f** Relative gene expression levels of *TCP22* (**d**), *RVE4* (**e**), and *LWD1* (**f**) in the wild-type (Col-0) and *che-2 Arabidopsis* plants at 24 hours after *Pst* DC3000 or *Pst* DC3000-HaRxL10 infection analysed by RT-qPCR with *UBQ5* as an internal control. The pathogen infection assay was conducted at ZT0. The data are shown as mean ± SEM (n = 9, 3 independent experiments with 3 technical replicates). The *p* values were calculated by two-way ANOVA followed by Holm-Šídák's multiple comparisons test.

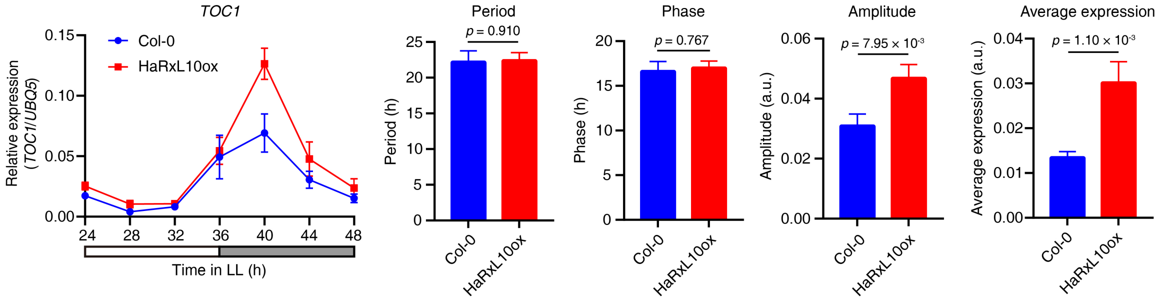

Supplementary Fig. 17 | HaRxL10 promotes *TOC1* expression. Relative expression levels of *TOC1* in 3-week-old wild-type (Col-0) and HaRxL10 overexpression (HaRxL10$_{ox}$ #18) *Arabidopsis* plants. Plants were grown under the 12 h light/12 h dark condition for 3 weeks and transferred to the constant light (LL) condition for 24 hours. Samples were collected every 4 hours under the LL condition and analysed by RT-qPCR with *UBQ5* as an internal control. White bar, subjective day. Grey bar, subjective night. The data are shown as mean ± SEM (n = 6, 2 independent experiments with 3 technical replicates). Period, phase, amplitude and average expression were calculated by nonlinear regression using a cosine wave. Data of period, phase, amplitude and average expression represent mean ± SEM (degree of freedom = 38). The *p* values were calculated by two-sided unpaired *t*-test with Welch's correction. This experiment was repeated twice with similar results.

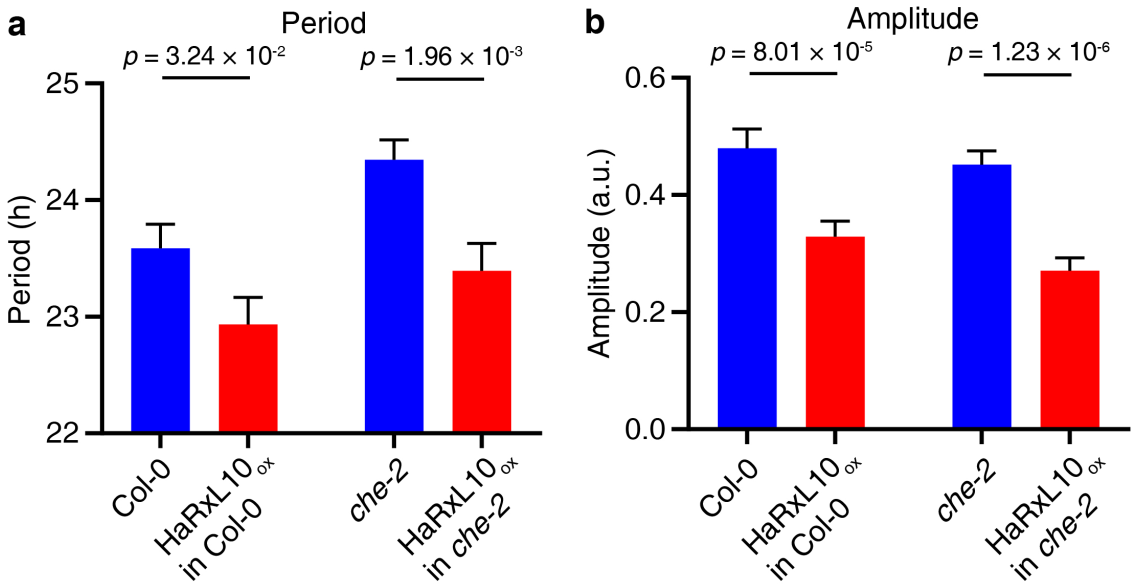

Supplementary Fig. 18 | HaRxL10 affects period and amplitude of leaf movement. Rhythm of leaf movement was measured in wild-type (Col-0) and HaRxL10 overexpression ($HaRxL10_{ox}$ #21 in Col-0) as well as *che-2* and HaRxL10 overexpression ($HaRxL10_{ox}$ #4 in *che-2*) *Arabidopsis* seedlings. Period (**a**) and amplitude (**b**) were calculated by nonlinear regression using a cosine wave. Data represent the mean ± SEM. The *p* values were calculated by two-sided unpaired *t*-test with Welch's correction. a.u., arbitrary unit. **Related to Fig. 7a, b.**

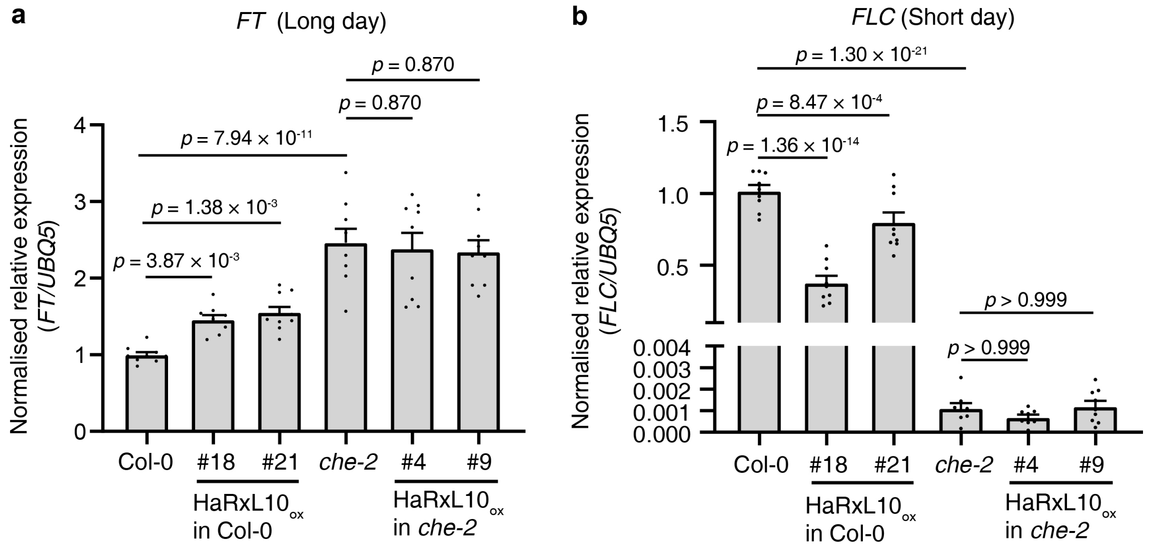

Supplementary Fig. 19 | HaRxL10 affects flowering gene expression in a CHE-dependent manner. Relative expression of *FT* (**a**) and *FLC* (**b**) in wild-type (Col-0), *che-2* and HaRxL10 overexpression (HaRxL10$_{ox}$) in different backgrounds (Col-0 or *che-2*) analysed by RT-qPCR with *UBQ5* as an internal control. The expression levels were normalised to the relative expression level in the wild-type plants. Long day, 16 h light/8 h dark. Short day, 8 h light/16 h dark. Ten-day-old seedlings were collected at ZT20 for *FT* expression analysis (**a**) and 5-week-old plants were collected at ZT (Zeitgeber time) 8 for *FLC* expression analysis (**b**). The data are shown as mean ± SEM (n = 9, 3 independent experiments with 3 technical replicates). The *p* values were calculated by one-way ANOVA followed by Holm-Šídák's multiple comparisons test.

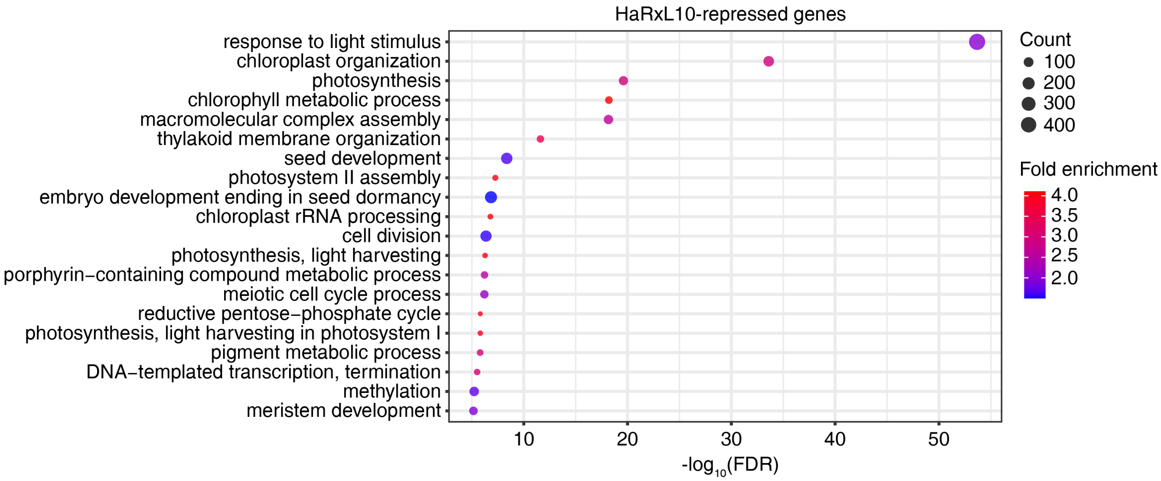

Supplementary Fig. 20 | Top 20 enriched GO terms of biological processes among HaRxL10-repressed genes. Top 20 enriched GO terms of biological processes among HaRxL10-repressed genes based on RNA-seq analysis of the wild-type (Col-0) upon *Pst* DC3000 and *Pst* DC3000-HaRxL10 infiltration. FDR, false discovery rate.

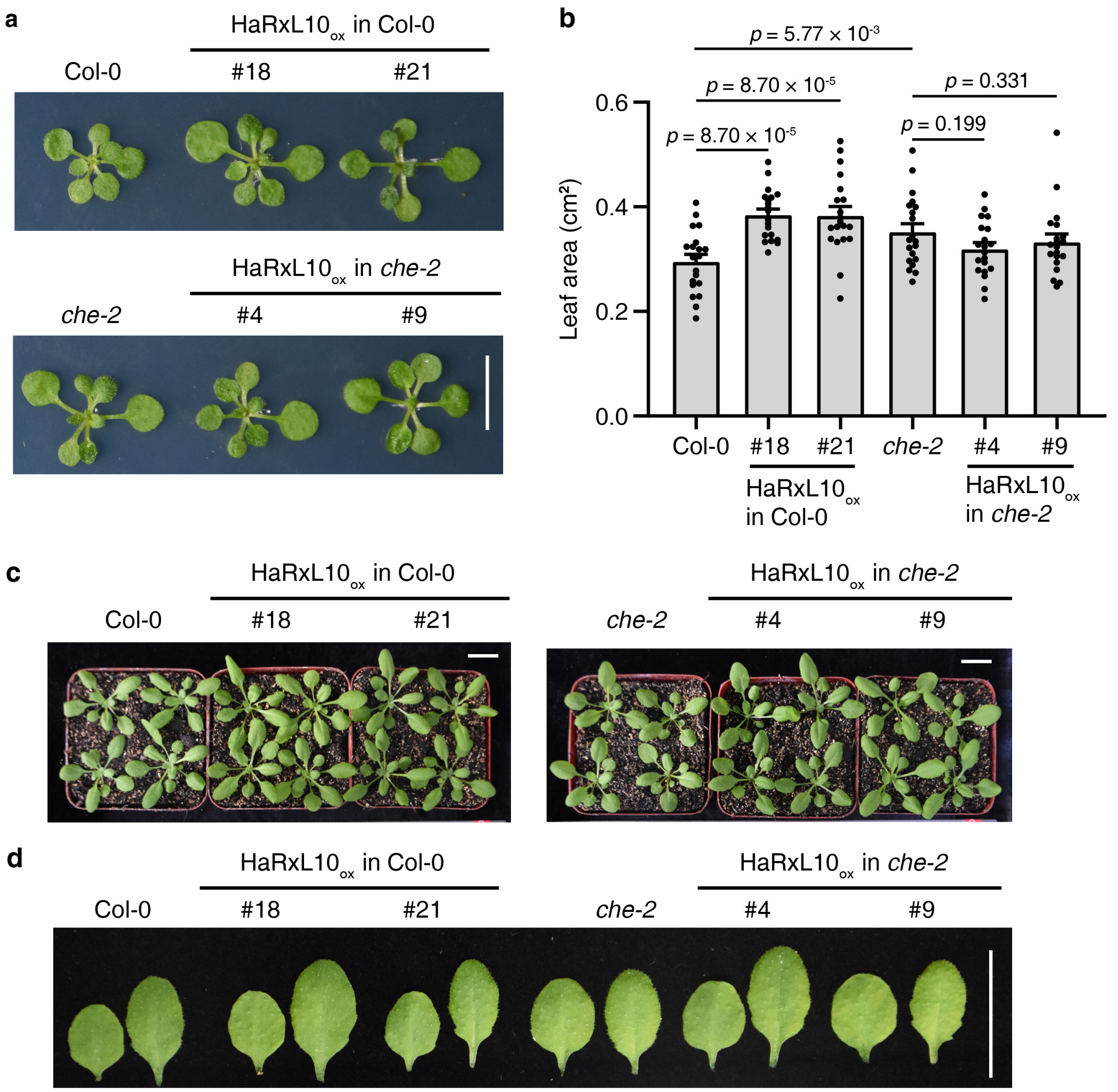

Supplementary Fig. 21 | The leaf phenotypes of HaRxL10 overexpression plants at different developmental stages. a Photographs of the representative 2-week-old *Arabidopsis* plants grown under the 12 h light/12 h dark condition. Scale bar, 1 cm. **b** The area of true leaves was measured from 2-week-old *Arabidopsis* plants growing under the 12 h light/12 h dark condition. The *p* values were calculated by one-way ANOVA followed by Holm-Šídák's multiple comparisons test. The data are shown as mean ± SEM (n = 20 leaves). **c, d** Photographs of 3-week-old *Arabidopsis* plants grown under the 12 h light/12 h dark condition (**c**) and their representative 4th and 5th leaves (**d**). Scale bar, 2 cm.

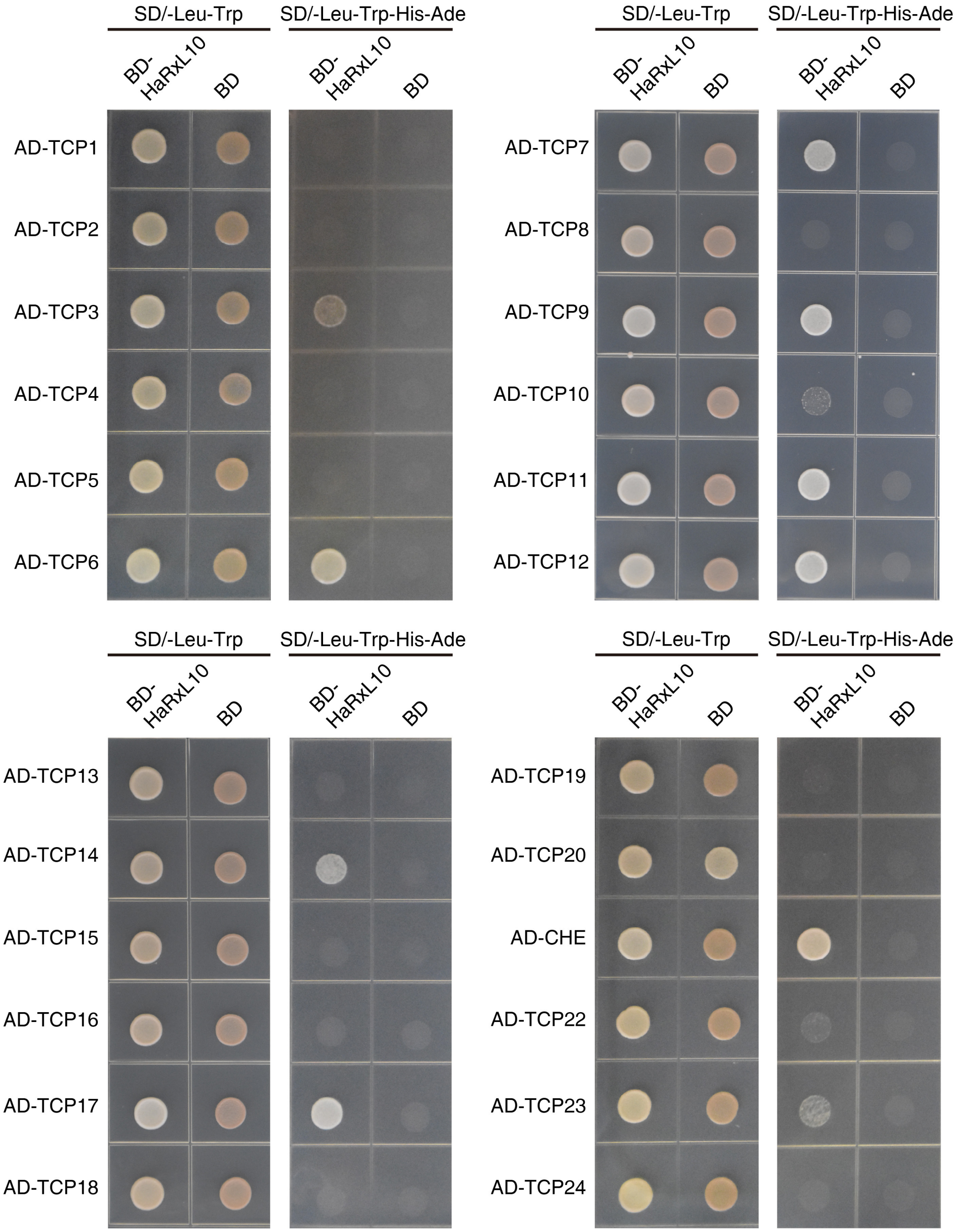

Supplementary Fig. 22 | Interactions between HaRxL10 and TCP family members in yeast. Interactions of HaRxL10 with TCPs in Y2H assays. Synthetic dropout medium without leucine and tryptophan (SD/-Leu-Trp) was used for positive yeast transformant selection. Synthetic dropout medium without leucine, tryptophan, histidine and adenine (SD/-Leu-Trp-His-Ade) was used for the selection of protein interaction by the reporter gene *HIS3*. Photographs were taken 2 days after plating of yeast cells with $OD_{600\ nm}$ = 1. This experiment was repeated three times with similar results.

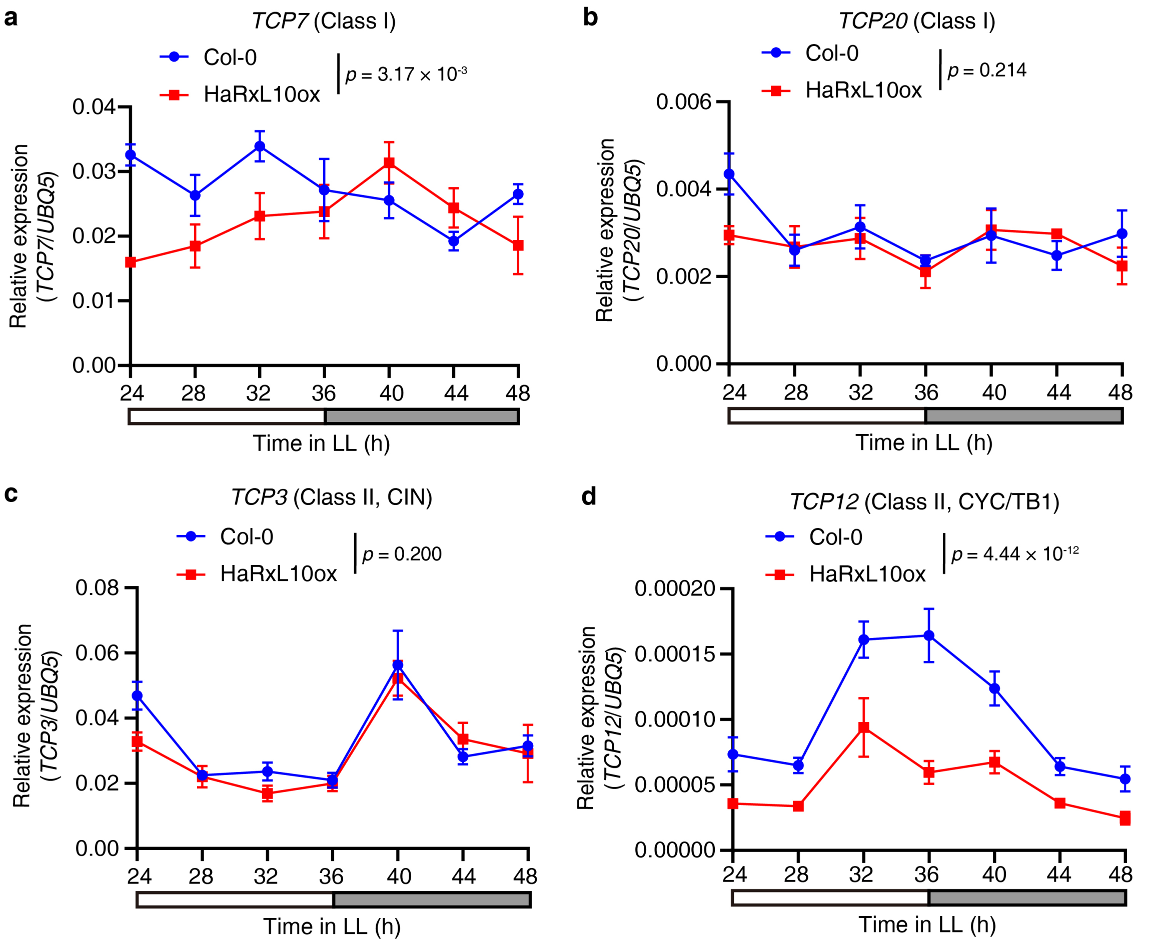

Supplementary Fig. 23 | Overexpression of HaRxL10 affects the expression of several *TCP* genes. a-d Relative expression levels of *TCP7* (**a**), *TCP20* (**b**), *TCP3* (**c**), and *TCP12* (**d**) in 3-week-old wild-type (Col-0) and HaRxL10 overexpression ($HaRxL10_{ox}$#18) *Arabidopsis* plants. Plants were grown under the 12 h light/12 h dark condition for 3 weeks and transferred to the constant light (LL) condition for 24 hours. Samples were collected every 4 hours under the LL condition and analysed by RT-qPCR with *UBQ5* as an internal control. White bar, subjective day. Grey bar, subjective night. The data are shown as mean ± SEM (n = 6, 2 independent experiments with 3 technical replicates). The *p* values were calculated by two-way ANOVA.

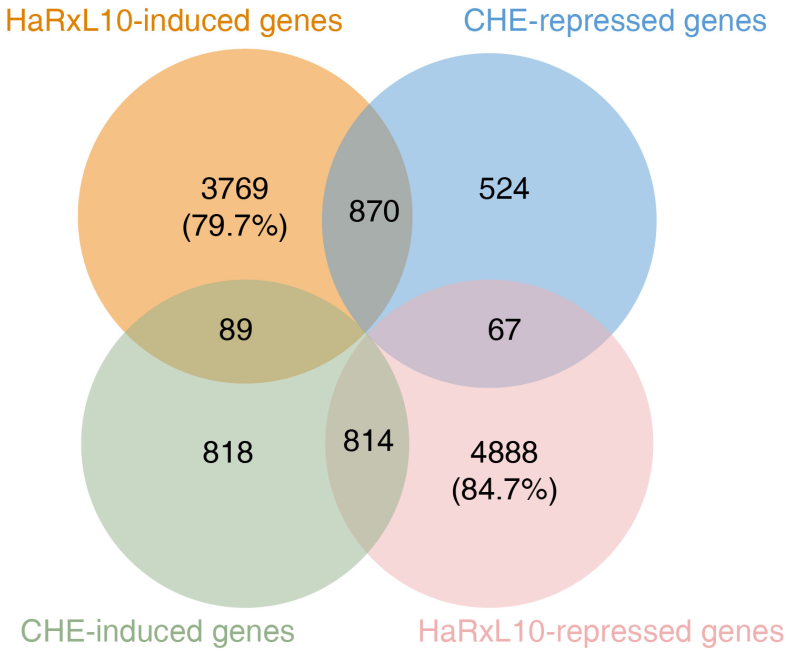

Supplementary Fig. 24 | Dependency of HaRxL10 responsive gene expression changes on CHE. Venn diagrams showing that 79.7% of HaRxL10-induced and 84.7% of HaRxL10-repressed genes are not dependent on CHE.
