# Supplementary material for: A pathogen effector HaRxL10 hijacks the circadian clock component CHE to perturb both plant development and immunity
Source: Nat Commun. 2025 Feb 11;16:1538. doi: 10.1038/s41467-025-56787-w (PMC11814294; doi:10.1038/s41467-025-56787-w)
Supplement: Supplementary file 2 — Description of Additional Supplementary Files [file 41467_2025_56787_MOESM2_ESM.pdf]

Description of Additional Supplementary Files:

Supplementary Data 1: Information of primers, materials, vectors, and sequences used in this study.

Supplementary Data 2: Information related to the time-course RNA-sequencing data.
